# Being born and growing up in the Ribeirão Preto and São Luís cohorts

**DOI:** 10.1590/1414-431X202111274

**Published:** 2021-03-04

**Authors:** M.C. Leal

**Affiliations:** 1 Departamento de Epidemiologia e Métodos Quantitativos em Saúde, Escola Nacional de Saúde Pública Sérgio Arouca, Fundação Oswaldo Cruz, Rio de Janeiro, RJ, Brasil

**Keywords:** Cohort study, Reproductive health, Risk factors, Breast-feeding, Caesarean, Maternal distress

## Abstract

This thematic issue consists of 14 articles derived from studies of the BRISA birth cohort (Ribeirão Preto, State of São Paulo and São Luís, State of Maranhão, northeastern Brazil, a socially and economically less developed region). In these more than 40 years of existence, these cohorts have been able to document the increase in women's education, the improvement of health conditions, the creation of a public Unified Health System (SUS) that provides universal and free access to health care, eradication of hunger, and transition of the nutritional status characterized by a decrease in malnutrition rates and an increase in obesity in Brazil. Particularly in reproductive health, the country experienced a significant drop in fertility, a decrease in maternal and child mortality, and an increase in breastfeeding rates. Universal access to prenatal care and hospital delivery was accompanied by an excessive number of cesareans without clinical indication and early-term births and premature births, largely due to scheduled cesareans. Articles with a longitudinal and transversal methodological approach are presented, using structural equation analysis and propensity score, together with multivariate regressions, which gave a robust analytical treatment to articles in this thematic issue.

## Presentation of Issue 54(1)2021

This thematic issue consists of 14 articles derived from birth cohort studies conducted in Ribeirão Preto (RP), State of São Paulo, southeastern Brazil, the most developed region in the country, and São Luís (SL), State of Maranhão, northeastern Brazil, the socially and economically least developed region. The articles cover a wide range of topics and methodological approaches. Started in 1978/79 in RP, the oldest birth cohort in the country had four additional waves: at 8-10 years of age, at 18 years evaluating only male individuals entering military service, at 23-25 years, and at 37-39 years. A second birth cohort was started in 1994 and the participants were evaluated at 8-11 years of age and, more recently, at 22-24 years. The SL cohort was started in 1997/98, with a second assessment at 7-9 years of age and, more recently, at 18-19 years of age. A first thematic issue reporting some of the results of these studies was published in this journal in 2007 (1).

In 2010, in an effort to further explore and expand aspects already addressed in the previous cohorts from the developmental origins of health and disease perspective, the RP and SL researchers simultaneously started a new birth cohort in both cities, called BRISA (Brazilian Ribeirão Preto and São Luís Birth Cohort Study). That study included all RP births and a sample of those born in SL in 2010, as well as a convenience cohort of pregnant women evaluated at 20-25 weeks of gestation and at birth in both cities. The mothers and their children were invited for a new assessment when the children were between 13 and 30 months old.

An interesting aspect of the cohorts is their composition involving two cities with different living conditions, which reflect the health conditions of the two places and which permit time-based assessment of the impact of federal public policies on these different social realities. During these more than 40 years of their existence, these cohorts have been able to document the major changes that occurred in the way of life and health standards of the Brazilian society: aging, increase in educational level especially among women, improvement of health conditions, creation of a public Unified Health System (SUS, in the Portuguese acronym) that provides universal and free access to healthcare, eradication of famine, and a nutrition transition characterized by a decrease in malnutrition rates and an increase in obesity. Particularly in the area of reproductive health, the main topic of the cohorts, Brazil has experienced a significant drop in fertility, a decrease in maternal and childhood mortality, and an increase in breastfeeding rates. Furthermore, the universal access to prenatal and hospital childbirth care was accompanied by an excessive number of cesarean sections without clinical indication and preterm and early-term births, resulting largely from scheduled cesarean sections (2–4).

Within the context of public health policies, a significant set of initiatives had an impact on these results, including the implementation of a national immunization program, programs combating diarrhea and acute respiratory infections, and programs for family planning, nutritional surveillance, smoking control, and breastfeeding promotion throughout the primary care network. In the hospital setting, the number of intensive care units was increased, especially neonatal units, and evidence-based procedures for the humanization of labor and childbirth healthcare were implemented (3).

The development of Science and Technology was another aspect of public health policies, enabled by SUS, which financed research and the production of supplies for the system and for society. The initiative boosted the production of knowledge in the area and supported experimental, clinical, and epidemiological studies involving the Brazilian population, as well as evaluative research of the programs implemented to identify barriers and facilitators. The partnership with national and state funding agencies for scientific and technological development in the country permitted the dissemination of this policy to all researchers that work in the different states of the federation (5,6).

The articles that are part of this thematic issue address several of the items mentioned above and can be analyzed from different perspectives: studies comparing outcomes among individuals of the same cohort over time; studies comparing outcomes in the two cohorts; etiological studies and studies of risk factors for the health of pregnant women and their newborns; etiological studies of outcomes in children born in different cohorts; studies of health service utilization; and studies classified by type and method of analysis. In addition, this thematic issue focuses on the importance of emotional factors as either determinants or consequences of the health condition of mothers and their children.

Nine articles had a longitudinal focus. Five of them addressed predictors measured during pregnancy and assessed outcomes at the time of delivery: pre-gestational BMI and its association with newborn weight (7); uterine artery pulsatility index and other maternal attributes such as risk factors for the development of gestational hypertension (8); measurement of cervical length, genitourinary infection, bacterial vaginosis, and prematurity (9); effect of genetic variants on the occurrence of intrauterine growth restriction (10); and consumption of soft drinks at 20-25 weeks of gestation, metabolic profile, and arterial hypertension (11). The other studies evaluated the long-term effects on the generations born during the cohorts: determinants of early weaning at less than 3 months and between 4 and 5 months of age (12); the importance of maternal cigarette and alcohol consumption during pregnancy and cognitive developmental delays in children after the second year of life (13); determinants of delayed first dental visit (not visiting a dentist before the age of 7) (14); and cesarean birth and mood disorders in adolescence (15).

The follow-up of a population, even over the short period of pregnancy, poses great challenges to the investigation because of the high turnover of families in the national territory and the changes of address within the same city, particularly in the case of the underprivileged population. Accompanying the population for analysis of long-term effects is truly an enormous task. These analyses provide important information that cannot be obtained in other types of studies and justify the maintenance of funding for the birth cohorts of the country. One factor not yet explored in the analyses are the consequences of preterm and early-term births for child health and development. Given the current model of obstetric care in Brazil, early-term births account for more than one-third of all deliveries and share some negative birth outcomes with preterm infants (16,17). What are the long-term consequences for these children? A combination of data from all cohorts may generate a sufficient number of infants born with this condition that would permit assessment of the effects on the future life of Brazilians. A partnership with the cohort of one hundred million Brazilians from the Center for Data and Knowledge Integration for Health (CIDACS), which compiles the routine data from the National Health Information Systems (18), would permit linking the databases of the RP and SL cohorts and CIDACS in order to carry out these analyses.

Four articles used cross-sectional analysis of the cohort data, three of them comparing the results obtained in the two cities: racial inequalities and their association with perinatal outcomes (19); comparison of the metabolic profile of pregnant women from the two cities (20); body image dissatisfaction of adolescents and symptoms of depression (21); and analysis of the association between maternal psychological distress and mother-child bonding only in SL (22). Finally, one article using data from SL validated a food frequency questionnaire for adolescents, qualifying its use in other studies (23).

Structural equation modeling and propensity score analysis, together with generalized linear models, permitted a robust analytical treatment of the data reported in the articles of this thematic issue in order to quantify risks and measure the strength of the associations between predictors and outcomes.

Finally, a comment should go on record here: the celebration of the launch of this thematic issue that addresses fundamental aspects of the reproductive health of the Brazilian population occurs at a time of great national suffering due to the occurrence of the Covid-19 pandemic that has already killed more than 242,000 citizens in the country (24), within a context of economic and social crisis and defunding of SUS and science and technology. We are appalled by the reintroduction of problems that we considered overcome such as hunger (25) and the control of vaccine-preventable diseases (26). Public health physicians and epidemiologists must highlight this in all texts they write during this period of consternation and sadness.
